# Overview of Machine Learning Process Modelling

**DOI:** 10.3390/e23091123

**Published:** 2021-08-28

**Authors:** Boštjan Brumen, Aleš Černezel, Leon Bošnjak

**Affiliations:** Faculty of Electrical Engineering and Computer Science, University of Maribor, Koroška Cesta 46, 2000 Maribor, Slovenia; ales.cernezel@gmail.com (A.Č.); leon.bosnjak@um.si (L.B.)

**Keywords:** data mining, machine learning, learning curves, learning process, power law

## Abstract

Much research has been conducted in the area of machine learning algorithms; however, the question of a general description of an artificial learner’s (empirical) performance has mainly remained unanswered. A general, restrictions-free theory on its performance has not been developed yet. In this study, we investigate which function most appropriately describes learning curves produced by several machine learning algorithms, and how well these curves can predict the future performance of an algorithm. Decision trees, neural networks, Naïve Bayes, and Support Vector Machines were applied to 130 datasets from publicly available repositories. Three different functions (power, logarithmic, and exponential) were fit to the measured outputs. Using rigorous statistical methods and two measures for the goodness-of-fit, the power law model proved to be the most appropriate model for describing the learning curve produced by the algorithms in terms of goodness-of-fit and prediction capabilities. The presented study, first of its kind in scale and rigour, provides results (and methods) that can be used to assess the performance of novel or existing artificial learners and forecast their ‘capacity to learn’ based on the amount of available or desired data.

## 1. Introduction

Ever since the advent of machine-stored data, there have been problems with the amount of data and the ability to store and process the data. In his seminal paper, E. F. Codd introduced the concept of relational databases because it was needed for “*protecting users of formatted data systems from the potentially disruptive changes in data representation caused by growth in the data bank and changes in traffic* [1].” Back in 1970s, Codd defined a “large” database as one having tables with 30 or more attributes.

Twenty years later, the concept of ‘data mining’ was introduced as a method of knowledge discovery in databases [2,3]. There was a general recognition that there is untapped value in greater collections of data and that such structures are indeed useful not only as repositories of atomic pieces of information, but rather that the database as a whole provides a lot of information, which can be used to guide business decisions and ultimately lead to a competitive advantage.

The general recognition was that novel approaches need to be implemented to mine the value from the data vaults. Typically, machine learning and artificial intelligence tools were employed. Researchers and practitioners examined various methods that were available and used them on a single dataset—the one they were trying to conquer. Very little research was done on the general applicability of these methods, which is why researchers were choosing the appropriate method by a trial and error approach. Once the problem was successfully solved, authors rarely investigated further possible improvements in a systematic way.

The possible improvements of a machine learner’s performance could come in several ways. Firstly, by improving and optimizing the algorithm itself. Secondly, by changing the internal parameters of a selected algorithm. Thirdly, the performance could be further improved by employing larger amounts of data. Ideally, the algorithm’s output could be analytically determined as a function of these three tactics. Different theoretical approaches provide estimates for the size of the confidence interval on the training error under various settings of the learning-from-examples problem. Vapnik-Chervonenkis (VC) theory [4] is the most comprehensive description of learning from examples. VC-theory provides guaranteed bounds on the difference between the training and generalization error. However, it has serious limitations, such as that it is applicable only to simple algorithms with a fixed ’capacity’, and requires an oracle that is never wrong. Hence, it was never used in real-life implementations. On the other hand, standard numerical (and other statistical) methods become unstable when using large datasets [5]. Theoretical approaches are unable to provide answers as to how learning algorithms learn with a given input, thus creating a research gap. Additionally, there are no methods developed to describe an algorithm’s performance on unseen data.

In this paper, we systematically explore the influence of the amount of data on the output of several machine learning algorithms and give a comprehensive description of their general performance. The research question is formulated as follows: (a) which of the models, in general, best describes the learning process of artificial learning algorithms, and (b) which of the models can most accurately predict the future performance?

The results of our study are significant for practitioners and developers of machine learning algorithms alike. Practitioners can use the results to verify if there is room for improvement of the generated model’s performance if more data were available and estimate the costs associated with additional data acquisition and preparation. Developers can use the methodology presented in this paper when they compare their novel algorithm’s performance with the existing ones in a systematic and rigorous way. To the best of our knowledge, the present study is the first one using several machine learning algorithms on such a large array of different datasets to obtain their performance envelopes.

The rest of the paper is organized as follows: the following section summarizes the existing research done in the field of learning curve approximation and prediction. In Section 3, the experimental setup is presented, while the results are discussed in Section 4. The paper is concluded with final remarks in Section 5.

## 2. Related Works

Mathematical descriptions of human cognitive abilities have already been the subject of substantial research. The idea behind this approach is based on the assumption that an existing mathematical function can be used to describe an individual learning curve, obtained from a given dataset. That is achieved by fitting the underlying parametric model to the learning curve in order to estimate it. Various mathematical functions have been studied extensively in literature in order to find the best parametric model to (a) interpolate the learning curve over the span of observed values, and (b) extrapolate the remainder of the curve beyond the range of known values.

Existing studies largely disagree on the most appropriate parametric model to describe and predict the learning process. Earlier studies often employed linear functions as benchmarks in their comparisons against other potential mathematical functions [6,7,8]. Although they were largely considered insufficient in their ability to describe the acquisition of new knowledge, there were nonetheless isolated cases in which they were demonstrated to provide the best goodness-of-fit. Logarithmic function was shown to be more promising. In [7], the best fit was achieved on four datasets. However, the measure was bound to the first portion of the learning curve, and was expected to perform worse for new points of data due to the function’s inflexibility.

The exponential function is often considered to be an established way of describing the acquisition of new knowledge [7,8,9]. As a result, the term *power law* has appeared [10]. In the last twenty years, however, some studies [11,12,13] have been suggesting that the power law arose as a result of averaging exponential curves. Using various simulations, the authors of the mentioned papers showed that if we monitor the progress of several students, their collective learning curve will be more similar to the power law, even if individuals learn according to the exponential law [11]. The same applies to non-trivial learning tasks, which can be divided into several differently demanding sub-tasks (i.e., when learning a foreign language, we are dealing with words and grammatical concepts of varying complexity). The mentioned research papers claim that while the progress of individual sub-tasks corresponds to the exponential law, the final progress of the entire learning task is in accordance with the power law, due to the effect of averaging.

In the existing literature on the machine learners, the power law has been most commonly considered the parametric model to offer the best fit. Frey and Fisher trained decision trees and found that on a total of 12 out of 14 datasets, the power law achieved the highest goodness-of-fit [6]. In [9,14], a three-parameter power function was compared to several simple and complex mathematical functions, and discovered that the former performed the best in most cases of comparison. Extended power law has been empirically shown to yield a well-fitting learning curve for the analysis of various parameters such as error and data-reliance in deep networks [15]. Recently, the power law has been employed for the purpose of learning curve fitting in the deep learning [16], natural language [17], medicine [18] and renewable energy domains [19].

However, other mathematical functions have also been successfully utilized in literature. Inverse power law was fit to a learning curve constructed on a small amount of data [20]. The authors then explored how well the estimated learning curve fit the entire learning curve on three large, imbalanced datasets, showing that the inverse power law is a suitable fitting method for big data. An exponential model has been used to follow and predict the spread of COVID-19 [21]. A weighted probabilistic learning curve model composed of several individual parametric models (including exponential and logarithmic) was empirically demonstrated to successfully extrapolate the performance for the purpose of deep neural network hyperparameter optimization [22].

Such cases suggest that the power law is not necessarily the most appropriate parametric model in all settings. The empirical evidence suggests that the choice may be dependent on the dataset and its properties, the classification learning method [23], the learning curve construction and fitting parameters, and other activities, such as pretraining and fine-tuning [15]. Generally speaking, the defined problem is the one that determines the shape of the learning curve; and while for most problems, it is possible to determine the best-fitting model, there are special cases for which the shape of the curve is difficult to characterize [24]. For the time being, there is no ideal parametric model that would be generally applicable in all situations, particularly in such cases as ill-behaved learning curves. However, it might be possible to identify a parametric model with a sufficient flexibility, and predictive ability [25].

One branch of research focuses on how a chosen parametric model can be adjusted in order to better fit the learning curve. For example, Jaber et al. improves the traditional power law model by taking into account the variable degree of memory interference that occurs across the repetitions that represent the learning-forgetting process [26]. In a study by Tae and Whang, a framework called Slice Tuner was proposed, which iteratively updates learning curves with acquisition of new data in order to improve model accuracy and fairness [27].

Another potential alternative approach is to empirically analyse the performance of an individual learning algorithm on as many datasets as possible. A series of statistical analyses can then be performed on the obtained results so that conclusions can be drawn from them. Frey and Fischer [6] measured the performance of decision trees and found that the shape of the learning curve can be described by the power law. Although many authors are in agreement with their findings [8,28], there are some that reject these claims [7]. In this paper, we improve and extend the empirical research carried out in [29]. The description, implementation and results of the experiment will be described in the following subsections.

## 3. Experiment

### 3.1. Experiment Design

As evident from the related work section of the paper, the two most commonly used models for describing learning curves are the *power* and *exponential* models. In [29], four models were used in total, namely the *power*, *linear*, *logarithmic*, and *exponential* models. However, the results showed that the linear model was not appropriate for describing the learning curve due to the basic shape of the linear function. Based on that, the linear model was excluded from this experiment. This experimental decision also reduced the overall complexity of the experiment, as fewer pairwise comparisons had to be conducted in the statistical analysis.

A few important improvements were introduced to the original experimental design [29]. The initial collection of datasets was expanded to 130 in total. The full list of datasets can be found in the Appendix A. The work in [29] focused on finding the best-fit learning curve for the C4.5 algorithm, which is a well-known implementation of decision trees. In this study, however, three additional classification algorithms were utilized: neural networks, Naïve Bayes, and support vector machines (SVM).

A more appropriate filtering of the constructed learning curves was also introduced. Since many learning curves were ill-behaved as a result of too fine granularity, a larger step increase had to be used. However, a coarser divide decreases the number of data points, which can be problematic when fitting learning curves constructed from smaller datasets. A balance between a fine and coarse divide was sought. The initial 10 instances [30] to be added to the next fold was shown to be producing too coarse learning curves, hence it was increased to 25. This choice reduced the number of ill-behaved learning curves to a minimum, while at the same time allowing for smaller datasets to be employed in the experiment. A more coarse divide into folds also resulted in a slightly lower computational complexity when generating the learning curves.

Next, a modified version of the coefficient of determination ${\bar{R}}^{2}$ was employed. The coefficient of determination $R^{2}$ measures the goodness-of-fit of a statistical model. Its value determines the proportion of variance of a dependent variable that can be explained or predicted by the independent variables. A higher value means a higher goodness-of-fit [31].

This coefficient, sometimes referred to as the *R*-square, is usually used to fit linear models, but it can also be used to fit nonlinear models. Depending on the purpose of use, the procedures for calculating its value also differ, and in some cases the value $R^{2}$ does not necessarily represent a square of a given value. Consequently, the values of this metric can also be negative.

Since different nonlinear functions were being fit with a different number of parameters, an adapted coefficient of determination ${\bar{R}}^{2}$, initially proposed by Theil [32], was chosen, instead. The equation for calculating the coefficient is as follows:

(1) $${\bar{R}}^{2}=1-\frac{SS_{res}/df_{e}}{SS_{tot}/df_{t}}$$

The values $df_{t}$ and $df_{e}$ represent degrees of freedom: $df_{t}=n-1$ and $df_{e}=n-p$, where *n* represents the number of instances in the population, and *p* represents the number of parameters of the fitted mathematical function (including the constant). The value $SS_{res}$ represents the sum of squares of residuals, and the value $SS_{tot}$ represents the total sum of squares. The two values can be calculated as:

(2) $$SS_{res}=\sum_{i}{\left(Y_{i}-\hat{Y_{i}}\right)}^{2}\;SS_{tot}=\sum_{i}{\left(Y_{i}-\bar{Y}\right)}^{2}$$

where $Y_{i}$ represents the actual value, $\bar{Y}$ represents the average of the actual values, and $\hat{Y_{i}}$ represents the predicted value within the given model.

The use of the mean square error (MSE) remained unchanged. The MSE is a measure for estimating the differences between the true value $Y_{i}$ and the predicted value $\hat{Y_{i}}$. It is defined as the mean of the square of the difference between the two values [33]:

(3) $$MSE=\frac{1}{n}\cdot \sum_{i=1}^{n}{\left(\hat{Y_{i}}-Y_{i}\right)}^{2}$$

The key difference between the metrics MSE and $R^{2}$ is that the former measures the exact deviation between the true and the predicted value, while the latter merely estimates the proportion of variance. It is recommended to use MSE for pairwise comparisons and statistical analyses, while $R^{2}$ is easier to understand and is more suitable for interpretation and presentation of the results.

Several changes were also made to the process of learning curve construction. The most important was the introduction of stratification. In this sampling method, the share of individual classes is calculated for the entire dataset. These proportions must then be maintained when creating subsets. Throughout the incremental addition of new instances, stratification ensures that each fold is a good representation of the entire dataset. It avoids uneven distribution of instances into classes, which can happen in some cases when random sampling is employed.

**Figure 1:**
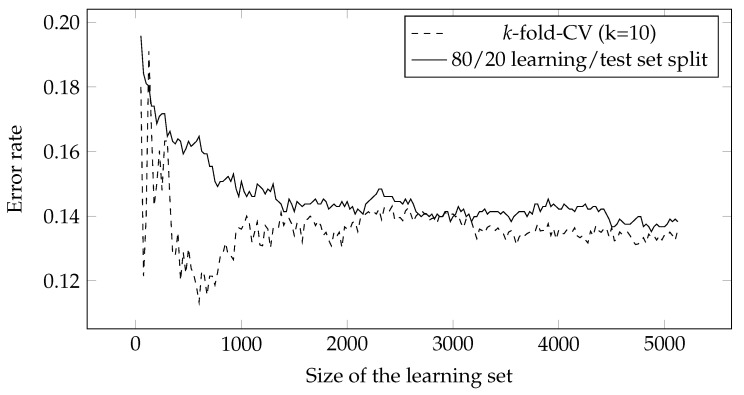
Comparison of methods for measuring accuracy in the construction of learning curves.

In order to measure the accuracy, each dataset had to be divided into learning and test sets. Earlier studies employed *k*-fold cross-validation [28,29,34], with the number of folds typically set to 10. This approach measures the error rate in a 10-fold run, and averages the result over all 10 folds. In this study, the datasets were divided in the 80/20 ratio. This is the simplest and least computationally demanding approach, which has also proven to be considerably more stable compared to the *k*-fold CV [35], as shown in Figure 1.

Individual learning curves were fitted to the following parameterized mathematical functions: power ($f_{pow}\left(x\right)$), logarithmic ($f_{log}\left(x\right)$), and exponential ($f_{exp}\left(x\right)$).

(4) $$f_{pow}\left(x\right)=p_{1}+p_{2}\cdot x^{p_{3}}$$

(5) $$f_{log}\left(x\right)=p_{1}+p_{2}\cdot log\left(x\right)$$

(6) $$f_{exp}\left(x\right)=p_{1}+p_{2}\cdot e^{p_{3}\cdot x}$$

**Figure 2:**
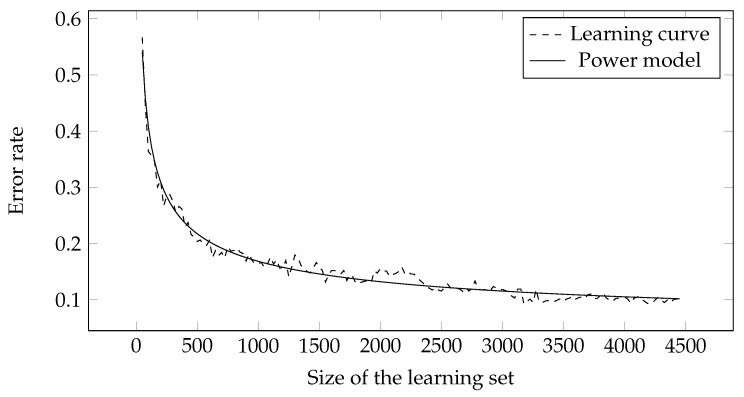
Fitting the learning curve to the power model.

It can be seen from the equations that the number of parameters $p_{i}$ differs between individual functions. All of them have the intercept parameter $p_{1}$ and the linear parameter $p_{2}$, while the exponential parameter $p_{3}$ is present only for the power and exponential functions. An example of fitting a learning curve with a power function is shown in Figure 2.

In terms of statistical analysis of data, more appropriate statistical methods were employed compared to [29]. Initially, the distribution of the data was verified using Kolmogorov-Smirnov and Shapiro-Wilk normality tests which showed that most datasets were not normally distributed. Instead of the classic *t*-tests and ANOVA, which assume normal distribution of data, we opted for their nonparametric alternatives, namely the Wilcoxon signed-rank test and Friedman’s test. We decided against using the Pearson’s $\chi^{2}$ test to determine the goodness-of-fit because this statistical test is not suitable for non-categorical data. Holm-Bonferroni correction was used instead of Bonferroni correction to correct for type I errors [36].

### 3.2. Experiment Execution

The first step in our experiment was to build the learning curves. Due to the large number of datasets and machine learning algorithms used, the learning curve construction process had to be fully automated.

For this purpose, a dedicated Java application that employed machine learning using the Weka Java API [37,38] was created. The application also took care of the preparation (stratification) and division of datasets into smaller (incremental) folds. An individual learning curve—for a specific dataset and a specific machine learning algorithm—was saved to a CSV file.

The construction of an individual learning curve was carried out according to the following procedure:

1. All instances in a given dataset are randomly rearranged.
2. The dataset is stratified before it can be divided in the 80/20 ratio.
3. The first 80% of the instances are separated from the main set to become the *learning set*. The remaining 20% of the instances comprise the *test set*.
4. All instances in the learning set are randomly rearranged.
5. The learning set is stratified before it can be divided into *k* folds. The number *k* is obtained by dividing the number of instances in the learning set by 25 and rounding the result down.
6. For each fold i∈{2,3,4⋯k}, the following is executed:
  (a) The first n=25·i instances are separated from the learning set and named the *learning subset*.
  (b) The selected classifier is trained on the learning subset.
  (c) The accuracy (Err) of the classifier is measured on the test set.
  (d) A pair of values (*n*, Err) is recorded.
7. All recorded values are saved to a CSV file.

After successfully creating all of the learning curves, the process of fitting the curves could begin. For this purpose, another dedicated Java application was developed so that the entire process could be fully automated. Apache Commons Mathematics Library was employed for this purpose. Their implementation of fitting nonlinear curves is based on the *Levenberg–Marquardt* algorithm, which works on the Least Squares principle [39].

Fitting of the individual learning curves was performed according to the following procedure:

1. The learning curve is read from the CSV file.
2. For each section of the learning curve i∈{1,2,3,4}, the following is performed:
  (a) All selected mathematical functions are fitted to the first ${}^{i}{/}_{4}$ points of the learning curve. The fit results are named $f_{pow}^{\left(i\right)}$, $f_{log}^{\left(i\right)}$ and $f_{exp}^{\left(i\right)}$.
  (b) For every fitted mathematical function ($f_{pow}^{\left(i\right)}$, $f_{log}^{\left(i\right)}$ and $f_{exp}^{\left(i\right)}$), the MSE and ${\bar{R}}^{2}$ are calculated for the first ${}^{i}{/}_{4}$ points of the learning curve. The results of these calculations are named $MSE^{\left(i\right)}$ and ${\bar{R}}^{2}{}^{\left(i\right)}$.
  (c) For every fitted mathematical function ($f_{pow}^{\left(i\right)}$, $f_{log}^{\left(i\right)}$ and $f_{exp}^{\left(i\right)}$), the MSE and ${\bar{R}}^{2}$ are calculated on the whole learning curve. The results of these calculations are named $MSE_{predict}^{\left(i\right)}$ and ${\bar{R}}^{2}{}_{predict}^{\left(i\right)}$.
  (d) For every fitted mathematical function ($f_{pow}^{\left(i\right)}$, $f_{log}^{\left(i\right)}$ and $f_{exp}^{\left(i\right)}$) the value vector ($MSE^{\left(i\right)}$, ${\bar{R}}^{2}{}^{\left(i\right)}$, $MSE_{predict}^{\left(i\right)}$, ${\bar{R}}^{2}{}_{predict}^{\left(i\right)}$) is derived.
3. All recorded values are saved to a CSV file.

It is apparent from the above procedure that each learning curve was fitted in quartiles, thus simulating the incremental addition of knowledge in four major steps. For each quartile, the metrics MSE and ${\bar{R}}^{2}$ were calculated twice. The first calculation was performed on the same points that were fitted, thus measuring the quality of the fit; the second calculation was performed on the entire learning curve, thus measuring the quality of the extrapolation of the learning curve (i.e., the prediction of the rest of the learning curve). Herein, it is necessary to point out that in the fourth quartile, the calculations are performed on the whole learning curve, which means that the prediction of the remaining learning curve was not feasible. In such cases, prediction could hypothetically be performed for scenarios in which we would like to know the future performance of the classifier if more data had been available. Based on that, it is possible to estimate the amount of data required to get the desired performance.

Due to the division into four quartiles, additional requirements regarding the choice of the learning curves were set. Each learning curve had to contain at least 20 points, or in other words—the dataset needed to have at least 500 instances in total. In this case, the learning curves in the first quartile would have at least five points, which is two more than the absolute minimum necessary to fit a mathematical function with three parameters (such as exponential and power functions). Due to this limitation, the number of datasets employed in the experiment ultimately varied between 79 and 130. The largest number of datasets was employed when an entire dataset was used for calculating the learning curve, without having to be split into quartiles (Filter = *none*).

Nonetheless, the incremental fit of learning curves in quartiles is not always successful. The algorithm used is not exact and may terminate in an error due to parameter limitations and exceeding the maximum number of iterations. As a result, the data to be used in the statistical analyses is further reduced. Because of that, the number of individual learning curves employed in statistical analyses was marked accordingly (column ’N’ in Tables 1 and 3). When no filter was applied (Filter = *none*), all 130 datasets were used.

However, when the filters were applied, some datasets did not have the required number of instances to produce enough data points for building the learning curve. We ended up with 79 datasets that provided enough data points for *all* filters, and provided answers for all four algorithms. Due to each mathematical model’s specifics, some calculations of models’ parameters diverged and no learning curve was produced. Such a case is presented in Table A3, for algorithm A in quartile 2 (see row 2), where power curve was not calculated. In some cases, we were unable to calculate any model for a specific algorithm. For example, when no filter was applied, there were a total of 130 datasets × 3 models = 390 potential learning curves. However, due to algorithms diverging and/or terminating, only 352 learning curves were successfully calculated. The datasets that did not evaluate one or more algorithms were used in the analysis in order to produce as many learning curves as possible, thus allowing multiple comparisons.

The complete data for the incremental fitting of learning curves for all algorithms and quartiles is given for a selected few datasets in the table in the Appendix B. The table shows the raw values of the metrics $MSE^{\left(i\right)}$, ${\bar{R}}^{2}{}^{\left(i\right)}$, $MSE_{predict}^{\left(i\right)}$, ${\bar{R}}^{2}{}_{predict}^{\left(i\right)}$. Missing entries indicate that the fitting was not successful for that configuration.

## 4. Results

In terms of fitting a single learning curve, the fit results of the selected mathematical models are interdependent. In other words, the results of the obtained metrics (MSE and ${\bar{R}}^{2}$) are interdependent within one learning curve and can be compared using pairwise (dependent) tests.

Since the obtained results do not satisfy the assumptions required for parametric tests, nonparametric tests in statistical analyses were used. Friedman’s test was employed for simultaneous comparison of all three mathematical models, followed by pairwise post-hoc tests using the Wilcoxon test of predetermined ranks.

Fitting the learning curves with different mathematical models was observed from two different perspectives. Initially, the goodness-of-fit, which shows how well a particular model can describe a part or the entirety of a learning curve that was examined. Then, its ability to predict, which shows how well a particular model can predict (or extrapolate) the remainder of the learning curve was investigated.

**Figure 3:**
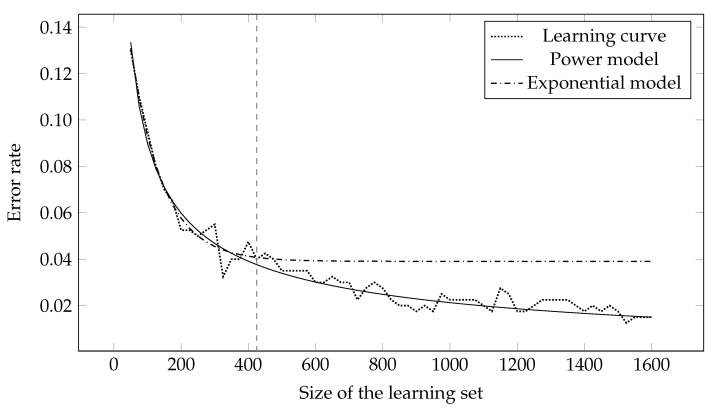
Fitting of the power and exponential model on the first quarter of the learning curve and extrapolation on the rest of the learning curve. The first quarter is marked by a vertical line.

Figure 3 shows the extrapolation of the learning curve using the power and exponential model. Both models were fitted on the first quarter of the learning curve, while the remainder was extrapolated—the milestone between interpolation and extrapolation is marked by a vertical line. It can be seen from the figure that the power model proved to be better at predicting the remainder of the learning curve.

### 4.1. Goodness of Fit

When comparing selected models in terms of goodness-of-fit, the $MSE^{\left(i\right)}$ was compared first, followed by ${\bar{R}}^{2}{}^{\left(i\right)}$. A more favorable value of an individual metric—lower $MSE^{\left(i\right)}$ and higher ${\bar{R}}^{2}{}^{\left(i\right)}$—means higher goodness-of-fit.

The Friedman test was used to compare $MSE^{\left(i\right)}$ of both models simultaneously. The results are shown in Table 1. Comparisons were performed for all four quartiles (Filter = *i*), as well as the full dataset (Filter = *none*). “N” represents the number of instances used in statistical comparisons. Due to limitations outlined in the previous section, the incremental fit of learning curves by quartiles was conducted on a limited number of instances. Conversely, the fitting on the full dataset was carried out on all available instances. Type I error corrections were performed for all five *p*-values in the table.

Due to the significant results of Friedman tests in the Table 1, post-hoc tests were required. Pairwise comparisons were performed using the Wilcoxon test of predicted ranks. The results of the pairwise comparisons of all three pairs are shown in Table 2. The table consists of five parts, which are separated based on the filter. The last column for every metrics marks the preferred model for describing the learning curves. If the *p*-value was not significant, the more appropriate model was not determined. Type I error corrections were performed for all 15 *p*-values in the table.

**Figure 4:**
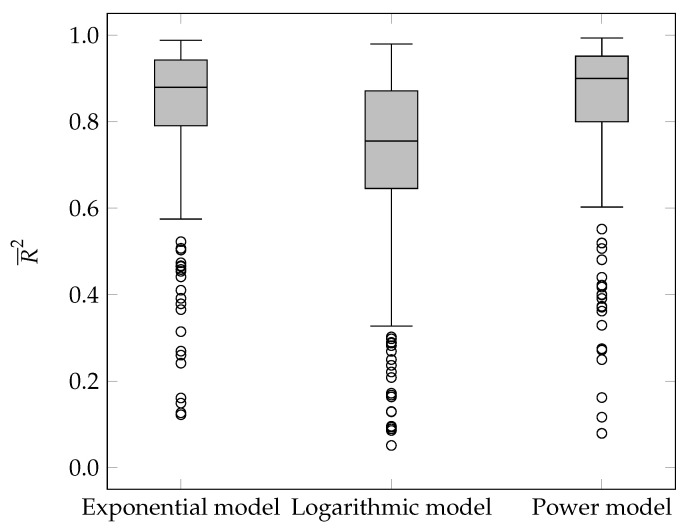
Statistical distribution of the ${\bar{R}}^{2}{}^{\left(i\right)}$ value.

After analyzing the $MSE^{\left(i\right)}$, we proceeded with analyzing the ${\bar{R}}^{2}{}^{\left(i\right)}$. Following the same procedure as before, the Friedman test was performed first, followed by pairwise comparisons using the Wilcoxon test of predicted ranks. The results of both statistical procedures are shown in Table 1 and Table 2. Since the value of ${\bar{R}}^{2}{}^{\left(i\right)}$ is generally restricted to the interval [0,1], the statistical distribution of values on the box-and-whisker plot were also shown (see Figure 4).

The power model had the highest median, followed by the exponential model, and finally the logarithmic model. With the exception of the first quartile (quartile=1), the power model proved to be the most appropriate. It is followed by the exponential, and finally, the logarithmic model.

### 4.2. Prediction

To compare mathematical models in terms of their prediction capabilities, a statistical analysis of the $MSE_{predict}^{\left(i\right)}$ and ${\bar{R}}^{2}{}_{predict}^{\left(i\right)}$ was performed. For both metrics, the model was initially fit on a part of the learning curve, and then measured its adequacy on the entire learning curve. This way, the basic model could be extrapolated. A more favorable value of an individual metric—lower $MSE_{predict}^{\left(i\right)}$ and higher ${\bar{R}}^{2}{}_{predict}^{\left(i\right)}$—means a greater ability to predict unknown data.

**Figure 5:**
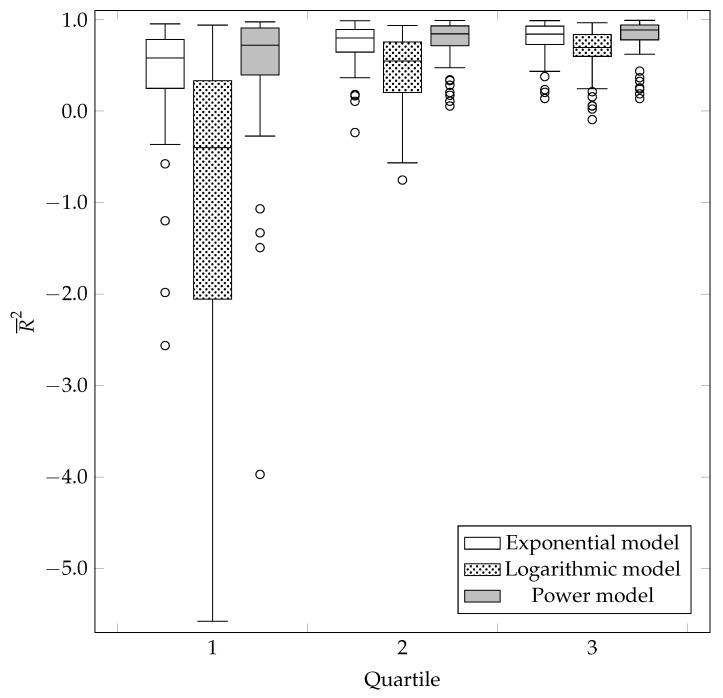
Distribution of ${\bar{R}}^{2}$${}_{predict}^{\left(i\right)}$ by quartiles.

The statistical analyses in this subsection are analogous to the ones performed in the previous subsection, so they were not described in more detail. The results of the Friedman comparison tests for $MSE_{predict}^{\left(i\right)}$ and ${\bar{R}}^{2}{}_{predict}^{\left(i\right)}$ can be found in Table 3. Table 4 contains pairwise comparisons of $MSE_{predict}^{\left(i\right)}$ and ${\bar{R}}^{2}{}_{predict}^{\left(i\right)}$ using the Wilcoxon predicate rank test. The statistical distribution of the ${\bar{R}}^{2}{}_{predict}^{\left(i\right)}$ for the selected mathematical models is shown on the box-and-whisker plot portrayed in Figure 5. Similarly to the goodness-of-fit measure, the power model had the highest median, followed by the exponential, and the logarithmic model.

With the exception of the first quartile (quartile=1) in the analysis of the metric $MSE_{predict}^{\left(i\right)}$, the power model again proved to be the most appropriate model for the prediction (extrapolation) of learning curves. It was followed by the exponential, and finally, the logarithmic model.

Since the model was fit to a portion of the learning curve, only the data for the first three quarters is shown in the mentioned figures and tables. That is because the fourth quartile represents the entire learning curve, for which any further predictions can no longer be validated using the existing data.

Type I error correction was performed on all three *p*-values for the Table 3, and all nine *p*-values for the Table 4.

## 5. Conclusions

When presenting the results from both aspects (fit quality and ability to predict), it was apparent that, in general, the *power model* proved to be the most appropriate choice for describing learning curves and thus machine learning algorithms’ performance. The results of the conducted research are consistent with the findings of authors in the area of machine learning, e.g., Frey and Fischer [6], Last [8] and Provost et al. [28].

Interestingly, the results contradict the findings of Heathcote et al. [11] who were modeling and observing human cognitive performance and found out that the exponential law is the best to describe an individual learner and that the power law may be observed only at the generalization level. However, the power law was again better at describing a combined motor-cognitive task [40]. There is additional research needed to explain why and when human and machine learners might be different in their performances.

The novelty of our research is in providing a systematic and concise answer regarding the shape of learning curves produced by artificial learning algorithms. No previous study has utilized a broad set of datasets and statistically validated the results. The studies mentioned here and in the related works have been working with mostly singlular machine learners and at best with a few datasets. As opposed to other studies, we have systematically investigated the performance and ability to predict of four commonly used machine learning algorithms over a substantial number of datasets, employing rigorous statistical methods.

The prevailing power law should be researchers’ first choice when measuring the performance of a learner at the individual level (a single machine learning algorithm) or at the generalized level (several algorithms). However, consistent with the observations of [15,23], a combination of decisions taken during the machine learning process (e.g., combination of datasets, selected classifiers, fitting parameters, pretraining, fine-tuning, etc.) determine the shape of the learning curve.

Our results can serve as important input to the practitioners who try to improve their results by changing the internal parameters of the machine learning algorithm used. The question for the practitioners is whether these changes lead to shifting the learning curve, or to a better generalization. Determining whether or not the change(s) affect the power-law exponent can lead to immense accuracy improvements. These can be implemented early in the process.

We have shown that for most problems it is possible to determine the best-fitting model and the best predicting model, but that there are special cases where the learning curve is difficult to characterize.

Future work should examine these cases in greater detail with the intention to identify and describe combinations of characteristics for which the power law is not the most suitable descriptor. A prominent area of the future studies is the impact of using data processing techniques (e.g., filtering, augmentation, cleaning) on the learning curves. Additionally, further studies should seek to find out which model is best for a specific algorithm.

## Figures and Tables

**Table 1:** Friedman’s test $MSE^{\left(i\right)}$ and ${\bar{R}}^{2}{}^{\left(i\right)}$.

		${\mathrm{MSE}}^{\left(i\right)}$			${\bar{R}}^{2}\left(i\right)$		
**Filter**	**N**	$\chi^{2}$	**df**	**Sig.**	$\chi^{2}$	**df**	**Sig.**
none	352	395.21	2	0.000	313.20	2	0.000
quartile = 1	228	89.40	2	0.000	55.50	2	0.000
quartile = 2	261	96.07	2	0.000	67.75	2	0.000
quartile = 3	276	105.80	2	0.000	100.78	2	0.000
quartile = 4	297	106.95	2	0.000	96.89	2	0.000

**Table 2:** Wilcoxon rank sum test—pairwise comparisons of $MSE^{\left(i\right)}$ and ${\bar{R}}^{2}{}^{\left(i\right)}$.

		${\mathrm{MSE}}^{\left(i\right)}$			${\bar{R}}^{2}\left(i\right)$		
**Filter**	**Pair**	***Z***	**Sig.**	**Best Model**	***Z***	**Sig.**	**Best Model**
none	log–exp	−12.76	0.000	Exponential	−13.77	0.000	Exponential
	pow–exp	−4.90	0.000	Power	−5.47	0.000	Power
	pow–log	−16.28	0.000	Power	−15.78	0.000	Power
quartile = 1	log–exp	−6.98	0.000	Exponential	−6.35	0.000	Exponential
	pow–exp	−1.50	0.133	—	−0.73	0.469	—
	pow–log	−7.62	0.000	Power	−6.89	0.000	Power
quartile = 2	log–exp	−6.13	0.000	Exponential	−7.10	0.000	Exponential
	pow–exp	−2.36	0.018	Power	−2.44	0.015	Power
	pow–log	−8.10	0.000	Power	−7.78	0.000	Power
quartile = 3	log–exp	-6.61	0.000	Exponential	−7.37	0.000	Exponential
	pow–exp	−2.59	0.010	Power	−3.32	0.001	Power
	pow–log	−8.33	0.000	Power	−8.24	0.000	Power
quartile = 4	log–exp	−6.10	0.000	Exponential	−6.73	0.000	Exponential
	pow–exp	−3.13	0.002	Power	−3.84	0.000	Power
	pow–log	−8.55	0.000	Power	−8.47	0.000	Power

**Table 3:** Friedman’s test $MSE_{predict}^{\left(i\right)}$ and ${\bar{R}}^{2}{}_{predict}^{\left(i\right)}$.

		*MSE* ${}_{\mathrm{predict}}^{\left(i\right)}$			${\bar{R}}^{2}{}_{\mathrm{predict}}^{\left(i\right)}$		
**Filter**	**N**	$\chi^{2}$	**df**	**Sig.**	$\chi^{2}$	**df**	**Sig.**
quartile = 1	228	72.34	2	0.000	74.00	2	0.000
quartile = 2	261	54.51	2	0.000	50.85	2	0.000
quartile = 3	276	74.11	2	0.000	68.94	2	0.000

**Table 4:** Wilcoxon rank sum test—pairwise comparisons of $MSE_{predict}^{\left(i\right)}$ and ${\bar{R}}^{2}{}_{predict}^{\left(i\right)}$.

		*MSE* ${}_{\mathrm{predict}}^{\left(i\right)}$			${\bar{R}}^{2}{}_{\mathrm{predict}}^{\left(i\right)}$		
**Filter**	**Pair**	***Z***	**Sig.**	**Best Model**	***Z***	**Sig.**	**Best Model**
quartile = 1	log–exp	−4.94	0.000	Exponential	−5.89	0.000	Exponential
	pow–exp	−1.63	0.104	—	−2.97	0.003	Power
	pow–log	−6.92	0.000	Power	−7.05	0.000	Power
quartile = 2	log–exp	−4.32	0.000	Exponential	−5.45	0.000	Exponential
	pow–exp	−3.51	0.000	Power	−3.66	0.000	Power
	pow–log	−6.95	0.000	Power	−7.16	0.000	Power
quartile = 3	log–exp	−5.42	0.000	Exponential	−6.18	0.000	Exponential
	pow–exp	−3.11	0.002	Power	−3.93	0.000	Power
	pow–log	−8.10	0.000	Power	−7.94	0.000	Power

## Data Availability

Publicly available datasets were analyzed in this study. This data can be found here: https://archive.ics.uci.edu/.

## Abbreviations

The following abbreviations are used in this manuscript:

AI	Artificial intelligence
ANOVA	analysis of variance
API	application programming interface
ARFF	Attribute-Relation File Format
CSV	comma separated values
ML	Machine Learning
MSE	mean square error
SVM	support vector machine
UCI	University of California Irvine

## Appendix A

The datasets used in our experiment were obtained from the UCI Machine Learning Repository [41]. There was a total of 184 datasets that focused on multivariate classification problems. However, some of the identified datasets were not appropriate for the task of constructing and fitting the learning curves, which is why they had to be removed. The primary criteria to determine the suitability of an individual dataset included: the number of instances, the availability of the dataset, and the format of the data.

If the dataset was already divided into learning and test sets, both sets were combined prior to the experimental procedure. Some of the published datasets were not dataset at all, but data created by random generators. Such cases were excluded as well.

Data format was also important. The tool that was used to implement machine learning, Weka, supports several forms of input data, however its native ARFF format proved to be the best for our purposes. Since the vast majority of datasets in the UCI repository were not available in this format, alternative solutions were sought. Several third-party repositories were found online, containing most of the collections from the UCI repository. The remaining datasets that could not be found in online repositories were manually converted. The few datasets that could not be converted to the desired format were discarded.

Finally, since datasets were split into quartiles, it was important to ensure that the learning curves contained enough data points. For that purpose, the required minimum number of instances in the collection was set to 500. The final number of datasets that met all the requirements was 79.

Table A1 displays a list of all datasets that provided enough data points for analyses to be conducted on individual quartiles. The meaning of the columns is as follows. The *Dataset* column indicates the name of the ARFF file, which in most cases matches the name of the dataset uploaded to the UCI repository [41]. The *Number of attributes* column indicates the number of attributes that represent potential decision criteria for classification. The *Number of instances* column marks the number of valid instances included in a given dataset.

**Table A1:** A list of 79 datasets from the UCI repository [41] that contained 500 or more instances.

Dataset	Number of Attributes	Number of Instances
ada_agnostic	49	4562
ada_prior	15	4562
analcatdata_authorship	71	841
analcatdata_dmft	5	797
analcatdata_halloffame	18	1340
anneal	39	898
anneal.ORIG	39	898
australian	15	690
balance-scale	5	625
breast-w	10	699
car	7	1728
cardiotocography	23	2126
CH	37	3196
cmc	10	1473
cps_85_wages	11	534
credit-a	16	690
credit-g	21	1000
csb_ch12	7	1601
csb_ch9	4	3240
cylinder-bands	40	540
diabetes	9	768
eucalyptus	20	736
eye_movements	28	10,936
genresTrain	192	12,495
gina_agnostic	971	3468
gina_prior	785	3468
gina_prior2	785	3468
HY	26	3163
hypothyroid	30	3772
ilpd	11	583
irish	6	500
jm1	22	10,885
kc1	22	2109
kc2	22	522
kdd_ipums_la_97-small	61	7019
kdd_ipums_la_98-small	61	7485
kdd_ipums_la_99-small	61	8844
kdd_synthetic_control	62	600
kropt	7	28,056
kr-vs-kp	37	3196
landsat	37	6435
letter	17	20,000
mammographic_masses	6	961
mc1	39	9466
mfeat-factors	217	2000
mfeat-fourier	77	2000
mfeat-karhunen	65	2000
mfeat-morphological	7	2000
mfeat-pixel	241	2000
mfeat-zernike	48	2000
mozilla4	6	15,545
MU	23	8124
mushroom	23	8124
nursery	9	12,960
optdigits	65	5620
page-blocks	11	5473
pc1	22	1109
pc3	38	1563
pc4	38	1458
pendigits	17	10,992
scopes-bf	21	621
SE	26	3163
segment	20	2310
sick	30	3772
soybean	36	683
spambase	58	4601
splice	62	3190
sylva_agnostic	217	14,395
sylva_prior	109	14,395
ticdata_categ	86	5822
tic-tac-toe	10	958
titanic	4	2201
train	15	5000
vehicle	19	846
visualizing_fly	2	823
vowel	14	990
waveform-5000	41	5000
wisconsin-diagnostic	31	569
yeast	9	1484

Additional analyses were carried out for learning curves that were constructed from the full datasets (i.e., when no filters were applied). Since the full datasets contained enough data points in all cases, the minimum number of instances requirement was not relevant. Table A2 lists the remaining 51 datasets that contained fewer than 500 instances. The analyses that were conducted on the datasets which were not split, employed *all* datasets listed in Table A1 and Table A2 (79 + 51 = 130 datasets).

**Table A2:** A list of 51 datasets from the UCI repository [41] that contained fewer than 500 instances.

Dataset	Number of Attributes	Number of Instances
analcatdata_braziltourism	9	412
analcatdata_broadwaymult	8	285
analcatdata_marketing	33	364
analcatdata_reviewer	9	379
arrhythmia	280	452
audiology	70	226
autos	26	205
badges_plain	2	294
baseball-hitter	24	322
baseball-pitcher	19	206
BC	10	286
Billionaires92	3	233
biomed	9	209
breast-cancer	10	286
cars_with_names	9	406
colic	23	368
colic.ORIG	28	368
credit	16	490
db3-bf	29	466
dermatology	35	366
ecoli	8	336
GL	10	214
glass	10	214
haberman	4	306
HD	14	303
heart-c	14	303
heart-h	14	294
heart-statlog	14	270
HO	23	368
ionosphere	35	351
jEdit_4.0_4.2	9	274
jEdit_4.2_4.3	9	369
kc3	40	458
liver-disorders	7	345
monks-problems-1_test	7	432
monks-problems-2_test	7	432
monks-problems-3_test	7	432
mw1	38	403
primary-tumor	18	339
prnn_fglass	10	214
prnn_synth	3	250
rmftsa_propores	5	289
schizo	15	340
seeds	8	210
sonar	61	208
spect	23	267
spectf	45	349
usp05	17	203
V1	16	435
VO	17	435
vote	17	435

## Appendix B

The Table A3 shows the experimental results of a comparison of eight selected mathematical models used to describe the shape of the learning curves. The meaning of the columns is as follows. The *Dataset* row preceding the tables and the *Algorithm* and *Quartile* columns show a combination of the selected dataset, the classification algorithm, and the size of the learning set. For each of the selected metrics ($MSE^{\left(i\right)}$, ${\bar{R}}^{2}{}^{\left(i\right)}$, $MSE_{predict}^{\left(i\right)}$ and ${\bar{R}}^{2}{}_{predict}^{\left(i\right)}$), the “Exp”, “Log” and "Pow” columns show the metric values for each quartile and selected mathematical models.

Algorithm legend: (A) decision trees: J48, (B) neural networks: Multilayer Perceptron, (C) Naïve Bayes: Naïve Bayes, and (D) support vector machines: SVM. Mathematical model legend: (Exp) exponential model, (Log) logarithmic model, and (Pow) power model.

**Table A3:** Comparison of selected mathematical models describing the shape of learning curves.

		${\mathrm{MSE}}^{\left(i\right)}$			${\bar{R}}^{2}{}^{\left(i\right)}$			$\mathrm{MSE}{}_{\mathrm{predict}}^{\left(i\right)}$			${\bar{R}}^{2}{}_{\mathrm{predict}}^{\left(i\right)}$		
**Algorithm**	**Quartile**	**Exp**	**Log**	**Pow**	**Exp**	**Log**	**Pow**	**Exp**	**Log**	**Pow**	**Exp**	**Log**	**Pow**
Dataset: *ada_prior*													
A	1	0.0056	0.0053	0.0053	0.0685	0.0165	0.0346	0.4736	0.5155	0.5065	−0.241	0.7041	0.3738
A	2	0.0078	0.0088		0.0682	0.0133		0.7629	0.7342		−0.235	0.7605	
A	3	0.0094	0.0099		0.0110	0.0129		0.8045	0.7952		0.8005	0.7680	
A	4	0.0102	0.0117	0.0116	0.0102	0.0117	0.0116	0.8159	0.7889	0.7894	0.8159	0.7889	0.7894
D	1	0.0012	0.0014	0.0008	0.0065	0.0766	0.0073	0.9085	0.9005	0.9432	0.7412	−2.025	0.7103
D	2	0.0019	0.0033	0.0012	0.0029	0.0241	0.0048	0.9102	0.8461	0.9425	0.8830	0.0473	0.8092
D	3	0.0024	0.0070	0.0022	0.0030	0.0094	0.0026	0.8957	0.6977	0.9024	0.8813	0.6272	0.8954
D	4	0.0029	0.0084	0.0026	0.0029	0.0084	0.0026	0.8837	0.6665	0.8957	0.8837	0.6665	0.8957
Dataset: *analcatdata_halloffame*													
A	1		0.0007			0.0051			−0.098			−0.310	
A	2		0.0015			0.0029			0.2211			0.2578	
A	3	0.0020	0.0020	0.0020	0.0029	0.0029	0.0029	0.2693	0.2977	0.2718	0.2345	0.2592	0.2407
A	4	0.0029	0.0029	0.0029	0.0029	0.0029	0.0029	0.2414	0.2686	0.2497	0.2414	0.2686	0.2497
Dataset: *car*													
B	1	0.0020	0.0028	0.0028	0.0183	0.1117	0.1047	0.9333	0.9180	0.9099	0.8465	0.0806	0.1209
B	2	0.0028	0.0070	0.0047	0.0209	0.0185	0.0061	0.9556	0.8948	0.9268	0.8249	0.8474	0.9491
B	3	0.0055	0.0087	0.0055	0.0085	0.0124	0.0058	0.9417	0.9112	0.9426	0.9283	0.8980	0.9515
B	4	0.0072	0.0108	0.0058	0.0072	0.0108	0.0058	0.9398	0.9114	0.9516	0.9398	0.9114	0.9516
D	1	0.0005	0.0022	0.0007	0.0191	0.1913	0.0158	0.9734	0.8998	0.9663	0.6635	−2.304	0.7225
D	2	0.0017	0.0111	0.0026	0.0302	0.0141	0.0256	0.9361	0.5910	0.9004	0.4674	0.7559	0.5487
D	3	0.0090	0.0138	0.0081	0.0198	0.0142	0.0143	0.7796	0.6724	0.8013	0.6508	0.7551	0.7479
D	4	0.0163	0.0141	0.0111	0.0163	0.0141	0.0111	0.7121	0.7563	0.8038	0.7121	0.7563	0.8038
Dataset: *csb_ch12*													
D	1	0.0007	0.0007	0.0006	0.0030	0.0209	0.0012	0.7996	0.8040	0.8158	0.6773	−1.162	0.8777
D	2	0.0009	0.0010	0.0007	0.0010	0.0071	0.0018	0.8693	0.8627	0.8945	0.8901	0.2601	0.8051
D	3	0.0010	0.0017	0.0009	0.0010	0.0031	0.0011	0.8877	0.8073	0.9002	0.8903	0.6770	0.8862
D	4	0.0010	0.0025	0.0010	0.0010	0.0025	0.0010	0.8903	0.7413	0.8936	0.8903	0.7413	0.8936
Dataset: *eye_movements*													
C	1		0.0077			0.1000			0.7201			−0.532	
C	2	0.0068	0.0099		0.0258	0.0599		0.8394	0.7677		0.6027	0.0818	
C	3	0.0151	0.0201	0.0199	0.0287	0.0514	0.0486	0.7616	0.6847	0.6852	0.5589	0.2117	0.2535
C	4	0.0236	0.0378	0.0338	0.0236	0.0378	0.0338	0.6368	0.4212	0.4806	0.6368	0.4212	0.4806
D	1	0.0049	0.0051	0.0037	0.1766	0.0123	0.0695	0.8208	0.8187	0.8644	−0.364	0.9054	0.4635
D	2	0.0122	0.0080	0.0077	0.0455	0.0134	0.0180	0.7874	0.8608	0.8654	0.6490	0.8971	0.8612
D	3	0.0148	0.0104		0.0177	0.0134		0.8623	0.9038		0.8634	0.8969	
D	4	0.0157	0.0122	0.0121	0.0157	0.0122	0.0121	0.8786	0.9058	0.9064	0.8786	0.9058	0.9064
Dataset: *genresTrain*													
A	1	0.0200	0.0142	0.0135	0.7900	0.0568	0.0321	0.9144	0.9398	0.9421	0.1544	0.9393	0.9656
A	2	0.0371	0.0194	0.0193	0.0969	0.0639	0.0548	0.9302	0.9638	0.9637	0.8963	0.9318	0.9414
A	3	0.0429	0.0288	0.0254	0.0669	0.0366	0.0280	0.9415	0.9608	0.9654	0.9284	0.9609	0.9700
A	4	0.0549	0.0340	0.0277	0.0549	0.0340	0.0277	0.9412	0.9637	0.9704	0.9412	0.9637	0.9704
C	1	0.0057	0.0060	0.0033	0.0174	0.1846	0.0248	0.6927	0.6792	0.8226	0.1473	−7.504	−0.144
C	2	0.0060	0.0128	0.0048	0.0066	0.0381	0.0107	0.6507	0.3569	0.7556	0.5267	−0.754	0.5068
C	3	0.0060	0.0177	0.0071	0.0060	0.0212	0.0082	0.5954	0.1677	0.6656	0.5692	0.0220	0.6212
C	4	0.0057	0.0198	0.0080	0.0057	0.0198	0.0080	0.5747	0.0896	0.6324	0.5747	0.0896	0.6324
D	1	0.0064	0.0066	0.0017	0.1963	0.1446	0.0124	0.9349	0.9336	0.9828	0.2269	0.4320	0.9512
D	2	0.0141	0.0102	0.0030	0.0393	0.0542	0.0043	0.9178	0.9407	0.9827	0.8451	0.7870	0.9830
D	3	0.0173	0.0161	0.0032	0.0231	0.0263	0.0037	0.9205	0.9265	0.9852	0.9091	0.8967	0.9856
D	4	0.0207	0.0219	0.0036	0.0207	0.0219	0.0036	0.9187	0.9140	0.9859	0.9187	0.9140	0.9859
Dataset: *gina_prior*													
C	1	0.0014	0.0015	0.0015	0.0096	0.0959	0.0879	0.8734	0.8708	0.8655	0.4589	−4.369	−3.971
C	2	0.0024	0.0042	0.0030	0.0036	0.0235	0.0085	0.8480	0.7384	0.8069	0.7954	−0.316	0.5199
C	3	0.0029	0.0071	0.0041	0.0034	0.0110	0.0051	0.8295	0.5947	0.7622	0.8101	0.3850	0.7092
C	4	0.0033	0.0092	0.0048	0.0033	0.0092	0.0048	0.8145	0.4838	0.7307	0.8145	0.4838	0.7307
Dataset: *kr-vs-kp*													
A	1	0.0037	0.0135	0.0027	0.0261	0.1384	0.0435	0.9012	0.6553	0.9286	0.1075	−0.843	0.4148
A	2	0.0118	0.0165	0.0064	0.0204	0.0511	0.0079	0.8002	0.7261	0.8911	0.7115	0.3189	0.8936
A	3	0.0145	0.0201	0.0069	0.0157	0.0289	0.0072	0.7903	0.7126	0.8997	0.7881	0.6152	0.9034
A	4	0.0153	0.0250	0.0072	0.0153	0.0250	0.0072	0.7940	0.6672	0.9034	0.7940	0.6672	0.9034
B	1	0.0022	0.0031	0.0029	0.0050	0.4181	0.2507	0.9620	0.9502	0.9510	0.9533	−2.847	−1.331
B	2	0.0024	0.0119	0.0054	0.0029	0.1028	0.0232	0.9730	0.8712	0.9403	0.9731	0.0537	0.7841
B	3	0.0027	0.0239	0.0079	0.0028	0.0399	0.0098	0.9731	0.7678	0.9224	0.9742	0.6331	0.9091
B	4	0.0028	0.0324	0.0089	0.0028	0.0324	0.0089	0.9743	0.7020	0.9172	0.9743	0.7020	0.9172
C	1		0.0049			0.0168			0.7999			0.8384	
C	2	0.0067	0.0083		0.0083	0.0211		0.8900	0.8672		0.9190	0.7966	
C	3	0.0071	0.0096	0.0093	0.0081	0.0141	0.0120	0.9171	0.8890	0.8913	0.9208	0.8638	0.8833
C	4	0.0081	0.0122	0.0107	0.0081	0.0122	0.0107	0.9213	0.8823	0.8959	0.9213	0.8823	0.8959
D	1	0.0006	0.0054	0.0007	0.0131	0.1433	0.0020	0.9763	0.7898	0.9699	0.6952	−2.293	0.9543
D	2	0.0016	0.0088	0.0009	0.0042	0.0319	0.0011	0.9537	0.7520	0.9745	0.9019	0.2661	0.9756
D	3	0.0023	0.0117	0.0009	0.0029	0.0163	0.0010	0.9428	0.7124	0.9763	0.9323	0.6250	0.9762
D	4	0.0028	0.0142	0.0010	0.0028	0.0142	0.0010	0.9361	0.6747	0.9762	0.9361	0.6747	0.9762

## References

[1] Codd E.F. (1970). A Relational Model of Data for Large Shared Data Banks. Commun. ACM.

[2] Piatetsky-Shapiro G., Frawley W.J. (1991). Knowledge Discovery in Databases.

[3] Fayyad U.M., Piatetsky-Shapiro G., Smyth P., Uthurusamy R. (1996). Advances in Knowledge Discovery and Data Mining.

[4] Vapnik V. (1982). Estimation of Dependences Based on Empirical Data.

[5] Dzemyda G., Sakalauskas L. (2011). Large-Scale Data Analysis Using Heuristic Methods. Informatica.

[6] Frey L., Fisher D. Modeling Decision Tree Performance with the Power Law. Proceedings of the Seventh International Workshop on Artificial Intelligence and Statistics.

[7] Singh S. (2005). Modeling Performance of Different Classification Methods: Deviation from the Power Law.

[8] Last M. (2007). Predicting and Optimizing Classifier Utility with the Power Law. Proceedings of the Seventh IEEE International Conference on Data Mining Workshops.

[9] Kolachina P., Cancedda N., Dymetman M., Venkatapathy S. (2012). Prediction of Learning Curves in Machine Translation. Proceedings of the 50th Annual Meeting of the Association for Computational Linguistics (Volume 1: Long Papers).

[10] Anderson J.R., Schooler L.J. (1991). Reflections of the Environment in Memory. Psychol. Sci..

[11] Heathcote A., Brown S., Mewhort D.J.K. (2000). The power law repealed: The case for an exponential law of practice. Psychon. Bull. Rev..

[12] Anderson R.B. (2001). The power law as an emergent property. Mem. Cogn..

[13] Murre J.M.J., Chessa A.G. (2011). Power laws from individual differences in learning and forgetting: Mathematical analyses. Psychon. Bull. Rev..

[14] Gu B., Hu F., Liu H. (2001). Modelling Classification Performance for Large Data Sets. Advances in Web-Age Information Management.

[15] Hoiem D., Gupta T., Li Z., Shlapentokh-Rothman M., Meila M., Zhang T. (2021). Learning Curves for Analysis of Deep Networks. Proceedings of the Machine Learning Research, Proceedings of the 38th International Conference on Machine Learning, Online, 18–24 July 2021.

[16] Hestness J., Narang S., Ardalani N., Diamos G.F., Jun H., Kianinejad H., Patwary M.M.A., Yang Y., Zhou Y. (2017). Deep Learning Scaling is Predictable, Empirically. arXiv.

[17] Kaplan J., McCandlish S., Henighan T., Brown T.B., Chess B., Child R., Gray S., Radford A., Wu J., Amodei D. (2020). Scaling Laws for Neural Language Models. arXiv.

[18] Kielaite-Gulla A., Samuilis A., Raisutis R., Dzemyda G., Strupas K. (2021). The Concept of AI-Based Algorithm: Analysis of CEUS Images and HSPs for Identification of Early Parenchymal Changes in Severe Acute Pancreatitis. Informatica.

[19] Hong S., Yang T., Chang H.J., Hong S. (2020). The effect of switching renewable energy support systems on grid parity for photovoltaics: Analysis using a learning curve model. Energy Policy.

[20] Richter A.N., Khoshgoftaar T.M. Learning Curve Estimation with Large Imbalanced Datasets. Proceedings of the 2019 18th IEEE International Conference On Machine Learning and Applications (ICMLA).

[21] Tuli S., Tuli S., Tuli R., Gill S.S. (2020). Predicting the growth and trend of COVID-19 pandemic using machine learning and cloud computing. Internet Things.

[22] Domhan T., Springenberg J.T., Hutter F. Speeding up Automatic Hyperparameter Optimization of Deep Neural Networks by Extrapolation of Learning Curves. Proceedings of the 24th International Conference on Artificial Intelligence.

[23] Guo H., Zhou J., Wu C.A. (2018). Imbalanced Learning Based on Data-Partition and SMOTE. Information.

[24] Vaitkevicius P., Marcinkevicius V. (2020). Comparison of Classification Algorithms for Detection of Phishing Websites. Informatica.

[25] Viering T., Loog M. (2021). The Shape of Learning Curves: A Review. arXiv.

[26] Jaber M., Peltokorpi J., Glock C., Grosse E., Pusic M. (2021). Adjustment for cognitive interference enhances the predictability of the power learning curve. Int. J. Prod. Econ..

[27] Tae K.H., Whang S.E. (2021). Slice Tuner: A Selective Data Acquisition Framework for Accurate and Fair Machine Learning Models. Proceedings of the 2021 International Conference on Management of Data.

[28] Provost F., Jensen D., Oates T. (1999). Efficient Progressive Sampling. Proceedings of the Fifth ACM SIGKDD International Conference on Knowledge Discovery and Data Mining.

[29] Brumen B., Rozman I., Heričko M., Černezel A., Hölbl M. (2014). Best-Fit Learning Curve Model for the C4.5 Algorithm. Informatica.

[30] Černezel A., Rozman I., Brumen B. (2014). Comparisons between Three Cross-Validation Methods for Measuring Learners’ Performances. Front. Artif. Intell. Appl..

[31] Glantz S.A., Slinker B.K. (1990). Primer of Applied Regression and Analysis of Variance.

[32] Theil H. (1961). Economic Forecasts and Policy.

[33] Lehmann E.L., Casella G. (1998). Theory of Point Estimation.

[34] Cohen P.R. (1995). Empirical Methods for Artificial Intelligence.

[35] Černezel A. (2016). Development of a Classifier Selection Method. Ph.D. Thesis.

[36] Abdi H., Salkind N.J. (2006). The Bonferonni and Šidák Corrections for Multiple Comparisons. Encyclopedia of Measurement and Statistics.

[37] Hall M., Frank E., Holmes G., Pfahringer B., Reutemann P., Witten I.H. (2009). The WEKA Data Mining Software: An Update. SIGKDD Explor. Newsl..

[38] Witten I.H., Frank E., Hall M.A. (2011). Data Mining: Practical Machine Learning Tools and Techniques.

[39] Levenberg K. (1944). A method for the solution of certain non–linear problems in least squares. Q. Appl. Math..

[40] Huang Y.T., Su Y.Y., Wu K.Y., Huang H.Y., Lin Y.S., Weng C.H., Yang L.Y., Pan Y.B., Wang C.J. (2020). Learning curve analysis of applying Seprafilm hyaluronic acid/carboxymethylcellulose membrane during laparoscopic hysterectomy. Sci. Rep..

[41] Lichman M., UCI Machine Learning Repository (2021). University of California, Irvine, School of Information and Computer Sciences. http://archive.ics.uci.edu/ml.

